# MED17/387: Development of a Mobile Internet-fitting Patient Monitoring System

**DOI:** 10.2196/jmir.1.suppl1.e60

**Published:** 1999-09-19

**Authors:** B Spyropoulos, M Botsivaly, C Coutsourakis

**Affiliations:** ^1^Technological Educational Institution of AthensAthensGreece

**Keywords:** Patient Monitoring, Decision Support

## Abstract

**Introduction:**

Setting-up a Monitoring System outside an Intensive Care Unit or even a Hospital, is often necessary or at least desirable. Therefore, a portable, multipurpose, quasi real-time, and fitting to the Internet Patient Monitoring System was developed.

**Methods:**

The system is composed of two PCs, equipped with data acquisition and A/D-converter boards, bio-signal preamplifiers, 2 Mbps wireless communication PCMCIA-Cards, and 56 K modems. Custom-made software is used for the formation and the handling of a Biosignal Knowledge Base. Variable length records of ECG, EEG and Respiration Rate, detected through the temperature variations, induced on a tailor-made thermo-couple sensor, can be acquired repeatedly and can appear directly on a proximal PC- display, or be transmitted through the wireless network to a distal PC, and be posted to a server. Beyond this quasi real-time patient monitoring procedure, the system can recognize Ventricular Tachycardia (VT) and Ventricular Fibrillation (VF), using a Threshold Crossing Intervals (TCI) technique. A threshold is set, so that the signal crosses it once per beat. VF, VT and Sinus Rhythms are characterized by distributions of threshold crossing, which can be identified, through statistical analysis techniques. Further, for the ECG classification and evaluation, the acquired signals can form each time a set of "patient vectors", which are compared to a set of "reference vectors", corresponding to cases, already evaluated by the medical expert, and constituting a continuously expandable knowledge base. An appropriate comparison metric allows for the retrieval of the "nearest" evaluated case, outfitting the system with a useful Decision Support Tool. The system allows for also EEG and other Biosignal processing.

**Results:**

Three data-sets were used to check the system, the first consisting of 100, 4 sec long records, generated by an ECG-simulator, combined to the data acquisition system, using a 250 Hz sampling frequency, the CSE multi-lead database, which includes 250, 10 sec long ECG records, and a set of 25 EEG and Respiration Rate records. Concerning VT and VF detection, the system is reaching an overall specificity of 96.9% and a sensitivity of 100%. The performance of the ECG search system depends on the acquisition conditions.

**Discussion:**

The system allows for the Acquisition, the Filing, the Processing and the Transmission through the Internet of ECG, EEG and Respiration Rate signals, either from patients or simulated cases, for diagnostic or educational purposes respectively. The system can be adapted to include Arterial Pressure and Cardiographic monitoring.

